# When Did They Start? Age of Onset of Toileting Behaviors and Urinary Cues as Reported by Older Women

**DOI:** 10.1089/whr.2023.0022

**Published:** 2023-07-28

**Authors:** Kathleen A. O'Connell, Diane K. Newman, Mary H. Palmer

**Affiliations:** ^1^ Department of Health and Behavior Studies, Teachers College Columbia University, New York, New York, USA.; ^2^ Perelman School of Medicine, University of Pennsylvania, Philadelphia, Pennsylvania, USA.; ^3^ School of Nursing, University of North Carolina, Chapel Hill, North Carolina, USA.

**Keywords:** female, aged, cues, urination, toileting behaviors, lower urinary tract symptoms

## Abstract

**Background:**

Lower urinary tract symptoms (LUTS) are prevalent across women's life course. Evidence shows toileting behaviors (TBs) and urinary urge cues are related to LUTS. It is unknown when women start using these behaviors and responding to urinary cues.

**Methods:**

An online survey was administered to 338 women, 65 years of age and older, to assess the age of onset for 20 TBs from the Toileting Behaviors-Women's Elimination Behavior (TB-WEB) questionnaire, 10 urinary urge cues from the Urinary Cues Questionnaire, and urinary urgency and leakage items from the International Consultation on Incontinence Questionnaire-Overactive Bladder (ICIQ-OAB) Short Form. Descriptive statistics were reported for each TB and urinary cue. A timeline was generated with the average earliest age of onsets for each type of TB, urinary cues, and urinary urgency and leakage symptoms.

**Results:**

Place preference, delayed voiding, and hovering over toilets away from home were reported to have the earliest ages of onset. Urinary urge cues, premature voiding, and straining to void tended to start after 45 years of age, as did the symptoms of urinary urgency and leakage. The timeline indicated that the earliest place preference and position preference TBs started before 20 years of age.

**Conclusion:**

Some TBs begin early in life and persist into old age, while other TBs and urinary cues begin later. Bladder health promotion may depend on intervening at specific times in the life course to alter TBs and responses, and potentially making environmental changes.

## Introduction

Lower urinary tract symptoms (LUTS) are prevalent across women's life course, are associated with co-morbidities, and are costly for women, the health care system, and society.^1^ Recent research has focused on bladder health (BH) promotion and LUTS prevention. This perspective that encourages investigation of factors outside the lower urinary tract system, which could protect or harm BH, led to the development of a bio-psychosocial conceptual framework^2^ and definition of BH by the Prevention of Lower Urinary Tract Symptoms (PLUS) Consortium.^3^

According to this conceptual framework, multilevel risk and protective factors must be considered from birth to old age.^2^ Congruent with this conceptual framework, women's BH was defined on a continuum as the self-report of no LUTS and no bladder interference in daily activities to almost always experiencing interference from LUTS.^4^ Secondary analyses of the Boston Area Community Health (BACH) baseline data from 3169 women between 30 and 80 years of age found that 20% were estimated to have optimal BH (*i.e*., no LUTS and no interference) and ∼20% reported poor BH (*i.e*., almost always LUTS and interference).^4^ Analyses from the BACH 5-year follow-up data revealed that a similar distribution of BH existed, reinforcing the notion that BH could be conceptualized as a spectrum.^5^

Earlier studies investigated the relationship between childhood and adult LUTS. One study followed women from birth to middle age. A positive association was found between mothers' reports of childhood enuresis at 6 years of age and the women's report of urinary incontinence in middle age.^6^ Another study was conducted with women between 40 and 69 years of age, who were asked to recall childhood bladder symptoms. Their reports of childhood LUTS, including urinary tract infections, were associated with symptoms in adulthood.^7^

Researchers also investigated the trajectories of bladder symptoms in earlier life stages. Five patterns of bladder symptom trajectories were identified in children (4 to 9 years of age), who were followed into adolescence.^8^ These patterns included normative development of day and nighttime bladder control, delayed attainment of bladder control, bedwetting alone, daytime wetting alone, and persistent wetting. Girls who had daytime wetting alone in childhood were more likely to have daytime wetting in adolescence. These findings support those from the studies with adult women that LUTS appear to persist over time.

This body of evidence indicates that further study of trajectories of LUTS and BH that includes bio-psychosocial and environmental factors is needed. The role some of these factors, including toileting behaviors (TBs) and urinary urge cues, play in relation to BH has been investigated.^9–11^ TBs include those behaviors women use before and during urination.^12^ These include behaviors related to place preference to urinate, premature urination, that is, voiding before a sensation of the need to void, delayed urination, that is, holding urine, while ignoring the sensation of the need to void, straining to void, that is, pushing or bearing down to start urinating, and position preference to urinate, for example, sitting on a toilet to urinate.

Conceptualized as part of Pavlovian processes, urinary urge cues are defined as conditioned stimuli or triggers individuals associate with the sensation of the urge to urinate, such as running water or arrival at one's door and cognitive processes, such as thinking of going to the bathroom. Earlier studies revealed that specific TBs, that is, delayed voiding, are related to specific health beliefs^13^ and women's reports of more urinary urge cues are related to bladder storage LUTS, that is, urinary urgency and urgency incontinence.^10,11^

Because urinary elimination is a lifelong function, behaviors and cues related to urinary elimination may be longstanding or may change with developmental stages, (*e.g*., adolescence). These behaviors may change in response to bodily conditions (*e.g*., pregnancy, menopause) and social influences (*e.g*., peers, employers, co-workers). The environment also plays a role in behaviors women use to urinate.^14^ Women often use different positions to urinate and have different levels of toilet access. Conditions of the toileting environment (*e.g*., cleanliness of the toilet facilities) may also play a role in behaviors women use in relation to urination.

In our previous work, we showed that (1) both toileting behaviors and urinary cues in women 65 years and over are related to current urinary symptoms, (2) toileting behaviors are related to urinary cues, and (3) these cues appear to mediate the relations between toileting behaviors and LUTS storage symptoms.^15^

Knowing when TBs and responsiveness to cues begin may be prerequisites to developing effective interventions. Behaviors and cues that arise early in life are likely controlled by different factors than those arising in young adulthood, or in menopause. The purpose of this article is to describe the age of onset of TBs and of urinary cues. We focused on women 65 years of age and older for several reasons, including TBs and urinary cues have been understudied in this population.^16^ Urinary symptoms are prevalent and often pose a significant problem for this population. Women in this age group provide an excellent vantage point from which to identify their own trajectory of behaviors and symptoms.

## Materials and Methods

### Study design

This is a descriptive study based on an online survey conducted in the Spring of 2020 with 338 women 65 years of age and older, unselected for urinary symptoms. Participants were members of panels of participants contracted by Qualtrics Research Services. Methods for the study, including recruitment, participants, and instruments were reported in a prior publication.^15^ The study was approved as exempt by the Institutional Review Board of Teachers College Columbia University. Of the 2043 respondents accessing the survey, 91% consented to participate. Of the 1856 who consented, 18.2% were included, 79.9% were not allowed to proceed with the survey because the quota for their race or ethnicity had been met, and 1.9% were excluded because they were not female or not 65 years of age or older, or because they finished the survey too quickly for their data to be considered trustworthy.

### Survey measures

Our instrument contained 136 items, including questions about demographic characteristics, items from the International Consultation on Incontinence Questionnaire Urinary Incontinence (ICIQ-UI) and a few other health-related items. For the analyses presented in this article, three different survey measures were used.

#### Toileting Behaviors-Women's Elimination Behavior Questionnaire

**FIG. 1.**
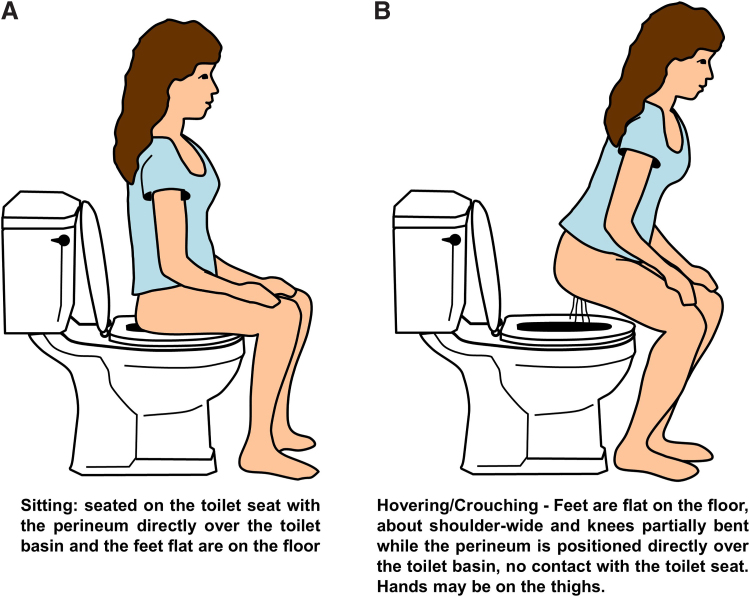
Illustration of toileting positions: **(A)** sitting. **(B)** hovering/crouching.

The Toileting Behaviors-Women's Elimination Behavior (TB-WEB) is a Likert-type questionnaire that consists of five domains: place preference (four items), premature voiding (five items), delayed voiding (three items), straining to void (the straining items specified “push down, strain/tighten my abdominal muscles” to begin urinating, to keep urine flowing, to empty bladder, and to empty bladder faster; four items), and position preference to void at home and away from home (six items). Position preference items were accompanied by a written and pictorial description of toileting positions, see Figure 1. Respondents have five response choices: never, rarely, sometimes, often, and always.^9^ The TB-WEB has been used in multiple female populations: college-aged students,^17^ health care professionals,^18^ employed women,^19^ and middle-aged and older women.^14^ It has been translated in multiple languages and used as an online and paper and pencil questionnaire.

#### Age of initiation of TBs

Follow-up questions to each of the TB-WEB items were used to assess participants' perceptions of when in their life course they started using various TBs. Response alternatives for these and other age-of-onset questions in this study were based on the stages of development identified by the PLUS Consortium researchers,^3,20^: before 11 years, 11–17, 18–25, 26–44, 45–64, and 65+ years. The age questions were skipped when the response to the initial question was “never.”

#### Age of onset of urinary cues

The 42-item Urinary Cues Questionnaire Version 2 was used to assess the stimuli the participants associated with the urge to urinate. Results for the cues items are reported elsewhere.^15^ To assess the age of onset of a subset of urinary cues, nine of the most frequently endorsed cues were selected on the basis of their item means in a prior study. The age of onset on one additional new cue was added: “After going to bed, but before going to sleep.” For these 10 cues, participants were asked: “To the best of your ability, estimate how old you were when you first noticed that the following situations increased your urge to urinate. If the situation did not increase your urge to urinate, please select Not Applicable (N/A).” Response alternatives were the same as those described above for the age of onset of TBs with the addition of the option, NA (not applicable).

#### LUTS measures

We also administered the International Consultation on Incontinence Questionnaire Overactive Bladder Short Form (ICIQ OAB-SF).^21,22^ After the item related to rushing to the toilet (urgency) to urinate, participants were asked, “How old were you when the need to rush to the toilet to urinate started?” After the item related to leaking before getting to the toilet, subjects were asked, “At what age did urine start to leak before you got to the toilet?” The age questions were skipped when the response to the initial question was “Never.” Response alternatives were the same as those described above for age of onset of TBs.

### Data analyses

Frequency and percentage of age of onset were calculated for the TB-WEB items, the Urinary Cues items, and the ICIQ-OAB rush and leak items. The frequencies and proportions of those who reported “Never” were also calculated. Figures were constructed using stacked bars showing the relative proportions of subjects reporting behaviors, cues, and symptoms at each age range and of those reporting “Never.” Then, we constructed a timeline of the age of onset for the variables studied.

To establish a timeline, earliest age of onset scores from each subscale of the TB-WEB, from the 10 urinary cues, and from the age of onset of the OAB symptoms of rush and leak were identified. Averages were computed from the scores of the earliest items in each subscale, which ranged from 1 (before 11 years) to 7 (75 or older). Subjects reporting “Never” were omitted from these analyses. Using the averages and the range that represented each score level, interpolations were carried out to generate a mean age of onset in years. These were placed on a timeline.

## Results

The sample consisted of 338 women who completed the survey. All women resided in the United States and read and spoke English. Their average age was 70.9 years (SD 5.55, range 65–97 years). The sample was recruited from the Qualtrics e-panel and women identified their race and ethnicity as follows: 22.5% identified as black persons, 67% identified as white persons, 5% identified as persons of other races, 3% of the sample identified as more than one race, and 2% preferred not to identify their race. Twelve percent (*n* = 39) identified as Hispanic persons. Twenty-six percent of the sample did not attend college and 34% had attended some college, while 39% had a bachelor's degree or above.

Approximately 84% of the sample (*n* = 285) reported having to rush to the toilet “occasionally” or more often in response to the ICIQ-OAB “rush” item. About 74% of the sample (*n* = 250) reported leaking before getting to the toilet “occasionally” or more often in response to the leak item on the ICIQ-OAB; 75% (*n* = 253) reported leaking urine at least “once a week or less often” in response to the leak question on the ICIQ-UI. Table 1 presents other results from the ICIQ-UI and other health-related items. The number of participants endorsing one or more of the 15 most common TBs ranged from 140 (41%) to 338 (100%). The number of participants endorsing one or more of the 10 urinary cues ranged from 139 (41%) to 226 (67%).

**Table 1.** Frequencies and Percents of Urinary Incontinence and Other Health-Related Variables *N* = 338

	Frequency	Percent
Frequency of urine leaks		
Never	85	25.1
About once/week or less often	120	35.5
Two or three times per week	49	14.5
About once a day	39	11.5
Several times a day	40	11.8
All of the time	5	1.5
Amount of leaks		
None	80	23.7
A small amount	228	67.5
A moderate amount	27	8.0
A large amount	3	0.9
Leak Interference with life (0 to 10)		
0	153	45.3
1–5	122	36.1
6–10	63	18.6
When does urine leak?^a^		
Never	71	21.0
Before you can get to the bathroom	198	58.6
When you are asleep	22	6.5
Physically active/exercising	62	18.8
When you are finished urinating and dressed	25	7.4
Leaks for no obvious reason	47	13.9
Leaks all the time	3	0.9
Need assistance to use the toilet?		
Yes	3	0.9
How would you rate your health?		
Excellent	23	6.8
Very good	95	28.1
Good	144	42.6
Fair	72	21.3
Poor	4	1.2
Body mass index^b^		
13.9–18.4 (underweight)	10	3.0
18.5–24.9 (healthy range)	108	32.0
25–29.9 (overweight)	88	26.1
30–39.9 (obese)	112	33.2
40 or over (severe obesity)	19	5.6
Incontinence treatments		
Kegel or pelvic floor exercises		
Yes	64	18.9
Over the counter or prescribed medicines		
Yes	23	6.8

^a^ Percents add to more than 100 because participant could check all that apply.

^b^ Data missing on one participant, *N* = 337.

### Toileting behaviors

**FIG. 2.**
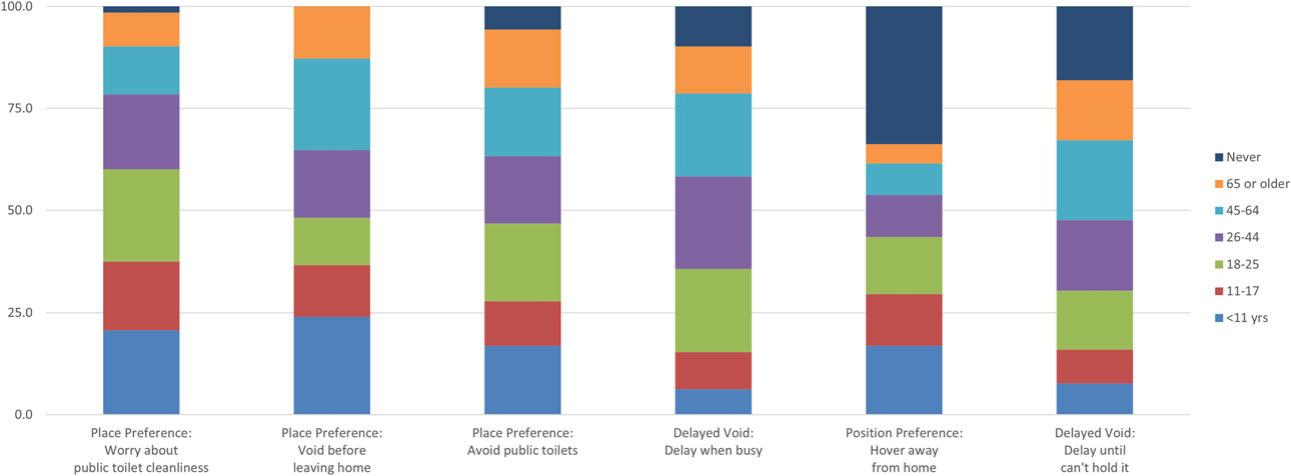
Age of onset of TBs that typically begin before 45 years of age. Like a pie chart, each bar shows the percentage of respondents who reported starting the behavior, while a certain age. For example, the first bar shows that about 20% of our respondents worried about public toilet cleanliness before they were 11 years old, while the second bar shows that even more, about 24%, began to void before leaving home by the same age (exact percentages are given in Table 1). Using bars rather than pies allows for easier comparisons. For example, the top of the purple section shows the percentage of our respondents who reported beginning a behavior before 45 years of age. The bars are sorted, left to right, by that before-age-45 percentage. The first bar shows that over 75% of our respondents had begun worrying about public toilet cleanliness by 45 years of age, but only about two-thirds had begun voiding before leaving home. The top section of each bar is the percentage of our respondents who reported never adopting the behavior. For example, about a third of our respondents reported never adopting the behavior of hovering when away from home (fifth bar). The other two-thirds began that behavior at the ages shown. TBs, toileting behaviors.

Table 2 displays the percent of all 338 participants adopting each TB for each age range and the percent who reported that they never engaged in TBs. Also included in Table 2 are the percent adopting each behavior before 45 years of age. The top portion of Table 2 shows the six TBs that were generally adopted before 45 years of age. These included three place preferences and two delayed voiding items and one position preference item. The onset of hovering over the toilet away from home was spread across the early years; 16.9% reported starting before 11 years of age, 12.7% between 11 and 17 years of age, and 13.9% between 18 and 25 years of age. Figure 2 displays this information in stacked bars with each age range represented by a unique color. Behaviors are presented in the order of cumulative percentages before age 45.

**Table 2.** Percentage of Participants Adopting Cited Behavior by Age in Years

	% <11	% 11–17	% 18–25	% 26–44	% 45–64	% 65+	% Never	% by 45
Behaviors usually adopted before 45 years of age								
Place preference: worry about public toilet cleanliness	20.7	16.9	22.5	18.3	11.8	8.3	1.5	78.4
Place preference: void before leaving home	24.0	12.7	11.5	16.6	22.5	12.7	0.0	64.8
Place preference: avoid public toilets	16.9	10.9	18.9	16.6	16.9	14.2	5.6	63.3
Delayed void: delay when busy	6.2	9.2	20.4	22.5	20.4	11.5	9.8	58.3
Position preference: hover away from home	16.9	12.7	13.9	10.4	7.7	4.7	33.7	53.8
Delayed void: delay until cannot hold it	7.7	8.3	14.5	17.2	19.5	14.8	18.0	47.6
Behaviors usually adopted at or after 45 years of age								
Premature void: unneeded void at home	4.7	4.1	7.1	8.6	23.7	26.0	25.7	24.6
Premature void: unneeded void just in case	5.6	4.1	6.8	7.1	26.0	22.2	28.1	23.7
Premature void: unneeded void away from home	3.0	5.0	6.2	9.5	20.4	20.1	35.8	23.7
Premature void: nnneeded void at another's house	2.7	2.1	3.6	10.9	16.0	18.0	46.7	19.2
Straining: push down to void faster	1.2	1.8	5.6	7.1	19.2	19.5	45.6	15.7
Straining: push down to finish voiding	0.9	2.1	5.6	6.2	18.0	22.8	44.4	14.8
Straining: push down to continue voiding	1.8	2.1	4.1	5.9	16.6	21.9	47.6	13.9
Premature void: void without need in public place	2.4	1.5	4.1	6.2	15.1	14.2	56.5	14.2
Straining: push down to begin voiding	2.1	1.5	5.0	5.3	12.7	14.8	58.6	13.9
OAB symptom: rush/urgency	6.2	1.2	1.8	3.6	33.7	37.9	15.7	12.7
OAB symptom: leak	2.7	0.3	1.2	3.0	23.1	43.8	26.0	7.1
Behaviors usually never adopted								
Position preference: straddle toilet seat away from home	1.8	5.0	5.0	3.0	2.4	2.4	80.5	14.8
Delayed void: delay at work	0.0	0.9	2.7	5.6	3.6	2.1	85.2	9.2
Position preference: hover over toilet seat at home	0.6	1.5	0.6	0.9	4.1	3.6	88.8	3.6
Position preference: straddle toilet seat at home	0.3	0.6	0.6	0.0	1.5	1.2	95.9	1.5

Each group is sorted by percent who have the behavior or OAB symptom by 45 years of age.

**FIG. 3.**
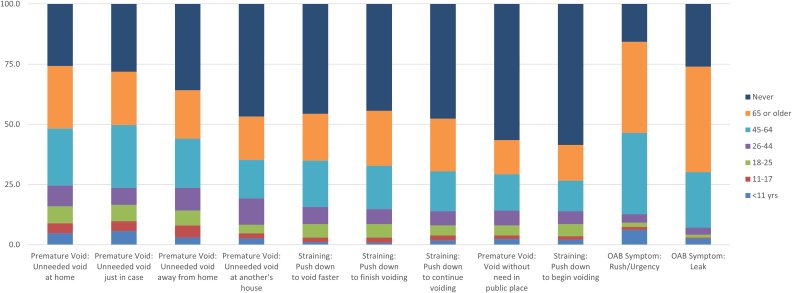
Age of onset of TBs that typically begin at or after 45 years of age and of urinary symptoms. See caption on Figure 1 for a guide to understand this figure.

The middle portion of Table 2 displays the nine behaviors generally adopted after 45 years of age. These include five premature voiding items and four straining items. For instance, the premature void item “Unneeded void at home” was adopted at or after 45 years of age by 49.7% of the women in the sample, while 24.6% reported adopting this behavior before 45 years of age, and 25.7% responded “Never” to this item. Also included in the middle portion are the ages of onset of the ICIS-OAB items for urgency and leaking. Figure 3 displays this information in stacked bars. The bottom portion of Table 2 lists the behaviors that were typically not adopted.

All TBs included in the survey were reported being used. Fifteen TBs were endorsed by more than 40% of the sample. Every woman reported voiding before leaving home with 48% reporting they began this behavior before they were 26 years old. While substantial proportions of subjects reported “Never” to straining items (44.4% to 58.6%), over one-third of the sample reported starting to engage in three of the four straining items at or after 45 years of age.

Also shown in both Table 2 and Figure 3 are the reported ages of onset of the LUTS symptoms of urinary urgency and leak. Approximately 84% of the sample reported urinary urgency, with 12.7% of the sample reporting onset of urgency before 45 years of age and 71.6% reporting age of onset at or after 45 years of age. Seventy-four percent of the sample reported leaking on the way to the bathroom, with 7.1% of the sample reporting onset of leaking before 45 years of age and over two-thirds (66.9%) reporting the age of onset at or after 45 years of age.

### Urinary urge cues

**FIG. 4.**
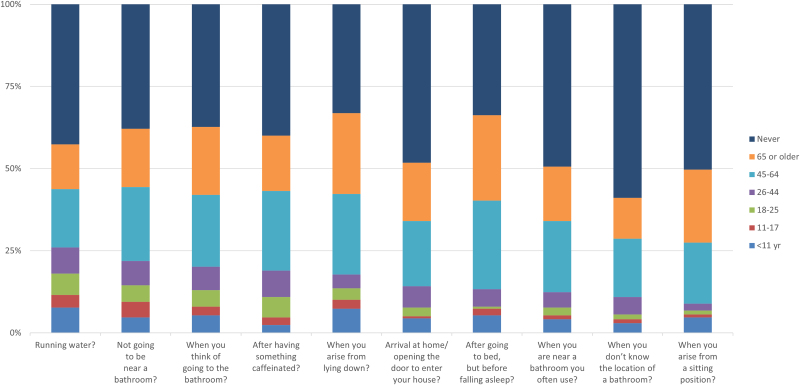
Age of onset of urinary urge cues. See caption on Figure 1 for a guide to understand this figure. For these data, exact percentages are shown in Table 2.

Table 3 and Figure 4 display the age of onset of urinary urge cues reported by women in the various age groups. In general, the age of onset of urinary cues did not start in women in younger years (<45 years). However, as expected, cues started to impact voiding behaviors in perimenopausal and aging women in significant numbers. Two types of cues were assessed: environmental cues and cognitive cues. One of the environmental urinary cues, running water, was reported to start before 45 years of age in 26% of the sample, but in 17.8% it started at 45–64 years of age and in 13.6% at 65+years of age.

**Table 3.** Percentage of Participants Reacting to Cited Cue by Age in Years

	% <11	% 11–17	% 18–25	% 26–44	% 45–64	% 65+	% Never	% by 45
Urinary urge cues								
Running water?	7.7	3.8	6.5	8.0	17.8	13.6	42.6	26.0
Not going to be near a bathroom?	4.7	4.7	5.0	7.4	22.5	17.8	37.9	21.9
When you think of going to the bathroom?	5.3	2.7	5.0	7.1	21.9	20.7	37.3	20.1
After having something caffeinated?	2.4	2.4	6.2	8.0	24.3	16.9	39.9	18.9
When you arise from lying down?	7.4	2.7	3.6	4.1	24.6	24.6	33.1	17.8
Arrival at home/opening the door to enter your house?	4.4	0.6	2.7	6.5	19.8	17.8	48.2	14.2
After going to bed, but before falling asleep?	5.3	2.1	0.6	5.3	26.9	26.0	33.7	13.3
When you are near a bathroom you often use?	4.1	1.2	2.4	4.7	21.6	16.6	49.4	12.4
When you do not know the location of a bathroom?	3.0	1.2	1.5	5.3	17.8	12.4	58.9	10.9
When you arise from a sitting position?	4.7	0.9	1.2	2.1	18.6	22.2	50.3	8.9

Each group is sorted by percent have the cue or trigger by 45 years of age.

The onset of the cue of “nearness to bathrooms” triggering a need to urinate started in later years, 21.6% in 45–64 years of age and 16.6% in 65+ years of age. The most frequent onset of the cue of caffeine ingestion was in women between 45 and 64 years of age at 24.3%. Women between 45 and 64 years of age (19.8%) and those 65+ years of age (17.8%) reported onset of the “key in the lock” cue, the need to urinate when arriving at home and opening the door.

### Timeline for ages of onset

**FIG. 5.**
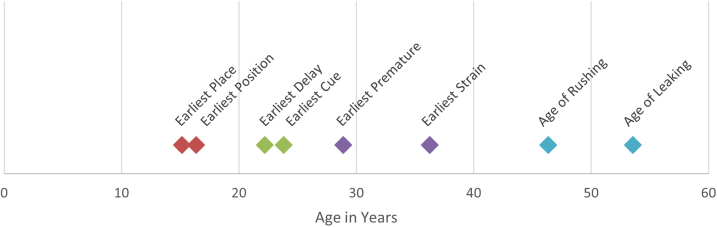
Timeline of mean age of onset by TB subscale, cues, and symptoms. For the TB subscales, first we determined each respondent's earliest age of onset for the items in that subscale. Earliest cue was determined by selecting the cue that had the earliest starting age. Points represent averages of earliest age of onset. The colors in this figure match the age groups in the other figures.

Figure 5 shows the timeline for the age of onset of the earliest TB in each subscale and of the earliest urinary urge cue and for rushing to the toilet and leaking urine. On average, the earliest place preference and position preference behaviors began in the mid to late teens. The earliest delayed voiding behavior occurred in the early twenties.

The earliest urinary cues were reported in the early twenties as well, suggesting that women begin connecting cues to the urge to urinate in early adulthood. The earliest premature voiding behavior started early in the adult premenopausal years (29 years of age), while the earliest straining began somewhat later in the premenopausal period (36 years of age). The LUTS storage symptoms occurred in perimenopausal period with feeling the need to rush to the toilet beginning around 46 years of age and urine leakage beginning, on average, around 53 years of age.

## Discussion

This survey study of 338 women, 65 years of age and older, assessed self-reports of age of onset of TBs, urinary urge cues, and the urinary symptoms of urgency and leakage. Place preference findings suggest that avoiding public toilets and using toileting positions, especially away from home, began early in the lives of these women. Hovering or crouching over the toilet when women were away from home was the major position preference variable endorsed in this sample.

There is little evidence about whether positions women assume to urinate affect bladder function. The limited research conducted to date has shown conflicting findings, but a recent study that included young nulliparous women found that uroflow parameters did not appear to be affected by sitting or hovering positions.^23^ The cumulative effect of the toileting position women use in their natural environment over their life course will be an important component to understand and improve women's BH. Women tend to worry about cleanliness of public toilets and engage in hovering over the toilet in order not to make direct contact.^24^ The concern over toilet cleanliness appears to occur across the life course.^16^

The onset of delayed TBs appeared early in adulthood and may coincide with the numerous roles, (*i.e.*, student, employee, parenthood), which tend to require women to be in environments that may not be amenable to voiding at will. The onset of premature voiding was predominantly after 45 years of age, about the same time that rush and leak symptoms appear. For some women, premature voids may be an adaptive response to prevent rushing to the toilet and leaking urine or to “keep the tank empty” so as to prevent embarrassment and bother from noticeable urinary leakage. These findings are similar to those reported by Newman et al.^14^

Although a small proportion of women report straining to urinate in childbearing years, perhaps due to pregnancy and pelvic organ prolapse, most reported the age of onset of straining to urinate in the perimenopausal and menopausal years. Thus, straining may be related to lower estrogen levels, aging, or unresolved problems of prolapse. It should be noted that women were not asked about age of onset unless they reported that they currently engaged in these behaviors. Thus, these early-onset behaviors have persisted for many years.

Cues associated with the urge to urinate may be more subtle than TBs. Individuals may not be aware of the effect of such cues until later in life. Earlier studies have shown that endorsement of cues is related to measures of LUTS in younger^25^ and older women.^15^ The results of this study suggest that the age of onset of most cues coincides with the age of onset of urinary urgency and leakage.

Women in this sample responded to our online survey in spring 2020, the beginning months of the severe acute respiratory syndrome coronavirus 2 (SARS-CoV-2) pandemic. Although women were not asked about their employment status, it was likely at the time of the survey that they were retired, not employed, or working from home due to the emerging SARS-CoV-2 pandemic.

Women with LUTS storage symptoms were not specifically targeted for recruitment in the study, although three quarters of the respondents reported having LUTS, specifically bladder storage symptoms of urgency and urgency incontinence at least occasionally or more often. A study by Abufaraj et al.^26^ showed that for women 60 years of age or older living in the United States, the estimated prevalence of urgency incontinence was 49.5%; the prevalence of mixed incontinence, which includes urinary urgency incontinence and stress incontinence, was 31.4%,^26^ yielding a combined prevalence of about 80%, which is consistent with the percent reported in this study.

Women who responded to the survey in 2020 were born between 1923 and 1955 with over 80% of the respondents born after 1945. Toilet training approaches varied over this period of time. For example, according to an infant care booklet for parents published in 1929, children should be able to control their bowels by 8 months and bladder training (*i.e*., offering the “chamber” on a frequent and regular basis and use of a cueing word) should be begun at 10 months, and the child should have daytime control by 18 months.^27^ During the 1940s and 1950s, Benjamin Spock, a pediatrician, advocated not starting toilet training before 2 years of age and allowing the child to show signs of readiness, develop familiarity with using the toilet, and starting to wear training pants once control is achieved.

How these methods of helping children meet the important developmental milestone of achieving continence affect acquisition of TBs and responsiveness to urinary cues is not known. However, the effects of toilet training methods may be reflected in our findings. One of the urinary cues, running water, is often used in toilet training as an audible signal that the child should try to emulate. The TBs of place preference and the position preference of hovering over a toilet when away from home start early in life and are consistent with a reluctance to urinate in public places. This reluctance was likely encouraged by parents' concern that the child would be exposed to diseases and children's own experiences and comfort using school toilets. Children report that they fear bullying and dislike smells in some restrooms.^28^

Around 8% of participants reported that they started to engage in premature voiding when they were quite young, perhaps incorporating the frequent entreaties from parents to empty their bladders, despite the absence of a conscious need to do so. Cues of nearness to bathrooms may also stem from early in life when parents encourage children to take advantage of available bathrooms whether they have a full bladder or not. It is possible that women who have abandoned these early behaviors and cues may return to them when bladder symptoms occur later in life. It may also be the case that TBs reflect women's attempts to remain continent in the event of changes to health, new developmental and social roles, and environmental constraints or challenges.

### Limitations

This study has a number of limitations. We are relying on women's memories of their TBs, urinary cues, and bladder symptoms. Validated instruments were used to assess current TBs, urinary cues, and OAB symptoms, but the age–of-onset questions pertaining to these items were new. Although we based our selection of age categories on those identified by the PLUS Consortium, and although the age categories coincide with major epochs in women's lives, the validity of responses may be affected by recall bias.

The ability of women accurately to place their experiences in these timeframes is unknown. We did not assess the onset of bladder symptoms other than rushing to the toilet and leaking urine associated with urgency incontinence. We did not assess the onset of stress urinary incontinence. One item from the place domain of the TB-WEB was inadvertently omitted from the survey. Other behaviors associated with urinary elimination, including, but not limited to, adjusting clothing before and after urination and speed of gait to the restroom, should be investigated.

Participation bias may be a limitation of this study. Respondents may be reluctant to participate because of embarrassment and stigma around urinary incontinence and other urinary symptoms. The anonymity of this survey may mitigate this factor. However, given the somewhat higher rate of leaking and urgency in this study compared to previous reports, it is possible that participants with LUTS may have been more inclined to participate than those without LUTS. Participants in the study panels recruited for the study are sent emails with list of studies they may be eligible for, but the topics of studies are not revealed until the participants access the survey and are presented with the study title in the informed consent form.

A total of 187 voluntarily discontinued participation after accessing the survey and finding out the topic. While this is a relatively large number, it represented only 9% of those who accessed the survey. Women without internet access would not have been invited to participate in our online survey. The study was conducted in the early months of the SARS-CoV-2 pandemic, which changed women's lives on multiple levels and could have affected their responses. At the end of the survey, we did ask women to tell us whether the pandemic affected their urinary behaviors. The respondents generally denied any effect. We present only descriptive statistics and do not have data to closely document the natural history of bladder symptoms in older women. Longitudinal studies are clearly needed.

## Conclusions

This report is among the first to document the age of onset of TBs and cues associated with urinary urges. Given that most TBs, urinary cues, and urinary symptoms start after 45 years of age, both perimenopausal and postmenopausal life stages would be excellent times to intervene to help women manage or prevent LUTS. Behaviors can be changed; cues can be extinguished. There is a possibility that simple awareness of problematic cues and behaviors may lead to their alteration.

To address behaviors and cues that start earlier in life, earlier use of the TB-WEB and the Urinary Cues Questionnaire may help identify problems before they lead to urinary symptoms. Place preference and premature voiding may be understandable responses to the lack of access and unclean public restrooms. Public health advocacy and health education are necessary to improve such access. Because of anatomical differences, restrooms for women need to be larger and somewhat more elaborate than urinals provided for men. Having access to safe, comfortable, and clean restrooms should be part of a women's rights agenda. However, access to safe and clean restrooms should be afforded to all regardless of gender. When clean restrooms are available, public health education should provide information about the problems of premature and unnecessary voiding, as well as delayed voiding and hovering over the toilet seats.

### Implications for future research

A bio-psychosocial conceptualization of BH suggests that more research is needed into the onset, persistence, and functionality of TBs that women employ. Some of these behaviors may serve important purposes in women's lives, but others may be related to decreased BH. Additional work on the relation of urinary urge cues to urinary urgency and urgency urinary incontinence could provide interventions to help women recognize and control their responsiveness to cues. New interventions, incorporating behavioral, psychological, and social factors, to promote BH and prevent urinary incontinence and other LUTS may be needed across women's life course as women lead their lives by engaging in multiple roles, activities, and environments.

## Abbreviations Used

BACH: Boston Area Community Health
BH: bladder health
ICIQ-OAB: International Consultation on Incontinence Questionnaire-Overactive Bladder Short Form
ICIQ-UI: International Consultation on Incontinence Questionnaire Urinary Incontinence
LUTS: lower urinary tract symptoms
N/A or NA: not applicable
PLUS: Prevention of Lower Urinary Tract Symptoms
SARS-CoV-2: severe acute respiratory syndrome coronavirus 2
TBs: toileting behaviors
TB-WEB: Toileting Behaviors-Women's Elimination Behavior
